# Less Severe Inflammation in Cyclic GMP–AMP Synthase (cGAS)-Deficient Mice with Rabies, Impact of Mitochondrial Injury, and Gut–Brain Axis

**DOI:** 10.3390/biology14111583

**Published:** 2025-11-12

**Authors:** Pannatat Areekul, Thansita Bhunyakarnjanarat, Sakolwan Suebnuson, Kollawat Somsri, Somchanok Trakultritrung, Kris Taveethavornsawat, Tewin Tencomnao, Siwaporn Boonyasuppayakorn, Asada Leelahavanichkul

**Affiliations:** 1 Department of Research and Development, Queen Saovabha Memorial Institute, Thai Red Cross Society, Bangkok 10330, Thailand; pannatattam@gmail.com (P.A.); Bank2701254@gmail.com (S.S.); 2 Center of Excellence in Translational Research in Inflammation and Immunology (CETRII), Faculty of Medicine, Chulalongkorn University, Bangkok 10330, Thailand; thansitadew@gmail.com (T.B.); kollawat.somsri@gmail.com (K.S.); 3 Department of Microbiology, Faculty of Medicine, Chulalongkorn University, Bangkok 10330, Thailand; somchanok29@gmail.com (S.T.); Kristaveethavornsawat@gmail.com (K.T.); siwaporn.b@chula.ac.th (S.B.); 4 Chulalongkorn University International Medical Program (CU-MEDi), Faculty of Medicine, Chulalongkorn University, Bangkok 10330, Thailand; 5 Center of Excellence on Natural Products for Neuroprotection and Anti-Ageing (Neur-Age Natura), Faculty of Allied Health Sciences, Chulalongkorn University, Bangkok 10330, Thailand; tewin.t@chula.ac.th; 6 Department of Clinical Chemistry, Faculty of Allied Health Sciences, Chulalongkorn University, Bangkok 10330, Thailand; 7 Division of Nephrology, Department of Medicine, Faculty of Medicine, Chulalongkorn University, Bangkok 10330, Thailand

**Keywords:** rabies, microbiota, macrophages, cGAS, mitochondria

## Abstract

Activation of cGAS, a receptor recognizing cytosolic DNA, in macrophages might be associated with rabies (an RNA virus) through mitochondrial damage. A similar mortality rate was observed between cGAS-deficient (cGAS-/-) and wild-type (WT) mice post-CVS-11 strain injection. However, 2 out of 12 cGAS-/- mice (but not WT) survived for 15 days post-injection. At 7 days post-infection, less severe brain inflammation in cGAS-/- mice was demonstrated by the viral abundance in the hippocampus, the expression of proinflammatory genes (*TNF-α* and *IL-1β*), and the Evans blue dye assay (blood–brain barrier defect) with the presence of higher anti-inflammatory genes (*TGF-β* and *arginase-1*). Fecal Proteobacteria was more prominent in the infected WT mice, while serum cytokines (TNF-α and IL-1β) were similar in both mouse strains. There were less prominent responses against the rabies virus in cGAS-/- macrophages than in WT cells, as indicated by supernatant IL-6 and the gene expression of *TLR-3*, *RIG-1*, *MDA-5*, and *iNOS*. On the other hand, mitochondrial injury and cGAS activation were more prominent in WT macrophages over cGAS-/- cells, as indicated by cGAS expression, supernatant cGAMP (a secondary messenger of cGAS), and mitochondrial oxidative stress (MitoSox) together with a decrease in mitochondrial DNA and maximal respiration (extracellular flux analysis). In conclusion, (i) rabies-damaged mitochondria led to cGAS activation that was less severe in cGAS-/- than in WT, (ii) rabies-induced dysbiosis was demonstrated, and (iii) cGAS manipulation and gut–brain axis-associated inflammation warrants further investigation.

## Simple Summary

Rabies is a deadly viral disease that attacks the central nervous system and causes death once symptoms appear. Despite being an RNA virus, rabies might be associated with the cytosolic DNA receptor, partly through mitochondrial DNA damage. Here, rabies in cGAS-deficient (cGAS-/-) mice was less severe than wild-type (WT) mice at 7 days post-infection, as indicated by viral burdens in hippocampus, blood–brain barrier defect, and inflammatory gene expression. Serum proinflammatory cytokines and gut permeability defect (FITC-dextran assay) in rabies-infected mice of both mouse strains were similar despite different fecal microbiome patterns. In parallel, cGAS-/- macrophages demonstrated less severe mitochondrial damage (MitoSox, mitochondrial DNA, and extracellular flux analysis) than WT cells after 24 h of incubation with rabies. Further studies on mitochondrial injury and the gut–brain axis in relation to rabies are needed.

## 1. Introduction

Rabies, caused by the rabies virus, a single-stranded negative-sense RNA virus belonging to the *Lyssavirus* genus within the *Rhabdoviridae* family, remains a significant global public health issue, particularly in Asia and Africa, with almost a 100% fatality rate once symptomatic [1]. The disease is transmitted primarily through the bites of mammals, especially dogs, and stray dogs are considered major carriers due to their close interaction with humans. However, other animals, including bats, foxes, wolves, and non-human primates, have also been identified as potential vectors. Rabies vaccination remains inadequate in many areas of the world, and the standard diagnostic method involves examining brain tissue for the virus [2]. Despite the importance of antibodies (the effectors of adaptive immunity), innate immunity is also important to control rabies virus [3]. After infection, the virus evades the host’s immune system and causes brain and central nervous system (CNS) inflammation through several recognition receptors, such as Toll-like receptor 3 (TLR-3; an endosomal receptor for dsRNA of the rabies virus during replication), TLR-7 (a receptor for ssRNA), and retinoic acid-inducible gene I-like receptors (RIG-I; a cytosolic receptor for most dsRNA and some ssRNA) [4]. Although rabies is an ssRNA virus, the cyclic GMP-AMP synthase (cGAS; a cytosolic DNA receptor) might be associated with the disease severity through the release of self-DNA [3] (mitochondrial DNA and host DNA) [5]. The rabies viral genome is packaged within a stable ribonucleoprotein (RNP) complex with the nucleoprotein (N), the phosphoprotein (P), and the RNA-dependent RNA polymerase (L) [6]. The P protein interacts with mitochondrial complex I (a critical component of the electron transport chain) that enhances superoxide radicals (an oxidative stress) of neuronal cells through an imbalance in electron flow [7]. Meanwhile, the matrix (M) protein surrounding the RNP core contributes to cell apoptosis [8]. Because rabies not only infects neurons but also invades microglia (macrophages in the brain) [9,10,11], the virus might also induce mitochondrial injury, increase free mitochondrial DNA (mtDNA) in the cell cytosol, and activate cGAS in macrophages. The harnessing of innate immune responses might be beneficial in rabies infection because macrophage neuroinflammation either reduces viral burdens (antiviral defense) or damages neurons [12]. Achieving balance within macrophage functions to limit damage with effective virucidal activity is important.

Although the roles of cGAS (a DNA receptor) and mitochondrial injury in dengue virus (a single positive-stranded RNA virus in the *Flaviviridae* family and genus *Orthoflavivirus*) [13,14] are mentioned, data on the impacts of cGAS in rabies are still limited. Despite both dengue and rabies being RNA viruses, both viruses differently affect mitochondria. While rabies virus (RABV) P protein directly binds with mitochondrial Complex I (a crucial enzyme in cellular respiration), leading to increased reactive oxygen species (ROS) production and mitochondrial dysfunction [15], dengue viral (DENV) proteins, for example, NS3 and NS4B, form physical contacts with mitochondria but do not directly bind to the mitochondrial matrix, causing mitochondrial elongation and fragmentation that disrupts ATP production and increases ROS levels [16]. Also, DENV inhibits mitophagy (a removal process of damaged mitochondria) that leads to the accumulation of damaged mitochondria and cell apoptosis [17]. During mitochondrial damage, mitochondrial DNA (mtDNA) is translocated from the mitochondria into the cytosol, and the DNA is recognized by several receptors in the cytosol, including cGAS [18]. The recognition of improperly located DNA, for example, DNA in the cell cytosol, leads to proinflammatory responses and cell damage [19]. Hence, cytosolic DNA might be involved in the pathogenesis of rabies, and thorough comprehension of this pathway would potentially enable a novel disease management approach.

Thus, the test of rabies infection using intramuscular (i.m.) injection of the virus in cGAS-deficient (cGAS-/-) mice and cGAS-/- macrophages compared with WT mice and macrophages is of particular interest. Moreover, the alteration in the gut microbiome, which influences the brain, referred to as the gut–brain axis (the bidirectional communication between the central nervous system (CNS) and the enteric nervous system), is another captivating aspect worth further exploring when concerning rabies encephalitis etiologies. The CNS influences gut motility, secretions, and immune responses through the autonomic nervous systems and the hypothalamic–pituitary–adrenal axis (via corticosteroids) [20]. Although bacteria do not have receptors for norepinephrine (a human hormone and neurotransmitter), some enteric bacteria respond to norepinephrine (catecholamines) from the hosts through bacterial adrenergic sensors (QseC, QseE, and RetS), triggering bacterial characteristics (growth, biofilms, and virulence) [21,22,23]. The alteration in gut bacteria during rabies infection might be associated with systemic inflammation, partly through gut permeability defects caused by dysbiosis (an imbalance of gut bacteria toward the pathogenic directions) [24]. Then, the immune response against rabies (an RNA virus) might involve recognition through the cGAS, a double-stranded DNA (dsDNA) receptor. We also explored fecal microbiome analysis with in vitro experiments using wild-type (WT) mice and cGAS knockout (cGAS-/-) mice.

## 2. Materials and Methods

### 2.1. Animal, Viral Preparation, and Animal Model

The Institutional Animal Care and Use Committee of the Queen Saovabha Memorial Institute (QSMI) under the Thai Red Cross Society, Bangkok, Thailand, approved the protocol (QSMI-ACUC-09-2025) according to the National Institutes of Health (NIH) criteria. Male wild-type (WT) C57BL/6 mice were purchased from Nomura Siam, Pathumwan, Bangkok, Thailand, while cGAS knockout (cGAS-/-) mice in the C57BL/6 background were kindly provided by Professor Søren Riis Paludan (Aarhus University, Aarhus, Denmark). The virus was prepared from the routine unit of the Department of Research and Development, QSMI, following the standard protocol [25]. To prepare the virus, the baby Hamster Kidney cell line (BHK-21) (Sigma-Aldrich, St. Louis, MO, USA) was grown in 20 mL of the supplemented Dulbecco’s Modified Eagle Medium (DMEM) in 10% fetal bovine serum (FBS) (Thermo Fisher Scientific, Waltham, MA, USA) at 37 °C with 5% CO_2_ in a culture flask until 80% confluence was reached, and BHK-21 monolayer was washed twice with sterile phosphate-buffer saline (PBS). Then, the challenge virus standard-11 (CVS-11; ATCC VR-959) (the American Type Culture Collection, Manassas, VA, USA) rabies virus [9] strain in multiplicity of infection (MOI) at 0.1 diluted in serum-free DMEM was added. Flask was incubated at 37 °C for 1 h and gently rocked every 15 min to remove the unbound virus prior to the addition of DMEM with 2% FBS. The cells were incubated at 37 °C with 5% CO_2_ and monitored daily for cytopathic effects (CPE) that can be visible within 24–72 h. When 80% CPE was observed, the supernatant was collected and centrifuged at 3000 rpm for 10 min. The virus-contained supernatant was aliquoted and stored at −80 °C for further experiments. To evaluate viral abundance in the supernatant, RNA was extracted from 60 µL of the preparation using the quantitative real-time polymerase chain reaction (PCR) following a published protocol [26]. Briefly, the TRIzol™ Reagent (Invitrogen, Thermo Fisher Scientific, Waltham, MA, USA) was utilized to extract total RNA from 25 mg of mouse brain tissue, and the quality of the extracted RNA was evaluated using a NanoDrop OneC Spectrophotometer (Thermo Fisher Scientific, Wilmington, DE, USA). For cDNA synthesis, 500 ng of isolated RNA was converted to cDNA using the High-Capacity cDNA Reverse Transcription Kit (Applied Biosystems, Thermo Fisher Scientific, Waltham, MA, USA). Each 20 µL reaction mixture contained 10 µL of the total RNA, 2 µL of 10× Reverse Transcription (RT) buffer, 2 µL of 10× RT Random Primers, 0.8 µL of 25× dNTP Mix (100 mM), 1 µL of MultiScribe™ Reverse Transcriptas (50 U/ µL), and 4.2 µL of Nuclease-free water. The PCR conditions were set as follows: 25 °C for 10 min, 37 °C for 120 min, and 85 °C for 5 min. The synthesized cDNA was subsequently stored at −20 °C for further analysis. The PCR was performed using a QuantStudio 6 Flex Real-Time PCR System (Applied Biosystems, Thermo Fisher Scientific, Waltham, MA, USA) with PowerUp™ SYBR™ Green Master Mix (Applied Biosystems, Thermo Fisher Scientific, Waltham, MA, USA). Each 10 µL PCR mixture contained 2 µL of cDNA, 5 µL of PowerUp™ SYBR™ Green Master Mix (2X), 0.2 µL of each forward and reverse primer (final concentration of 1 µM), and 2.6 µL of DNase-free water. The primer sequences are listed in Table 1. The thermal cycling conditions were as follows: the initial temperature was 50 °C for 2 min, followed by denaturation at 95 °C for 10 min, and then 40 cycles of denaturation at 95 °C for 15 s, annealing at 60 °C for 60 s, and extension at 95 °C for 15 s, followed by 60 °C for 60 s and 95 °C for 15 s. The abundance of virus was reported in “viral copies” determined from 60 µL of viral preparation.

Then, the intramuscular inoculation of the viral reparation or diluent control was conducted in 4-week-old mice at both hind limbs (30 µL per site; total virus approximately at 8 × 10^9^ copies). The dose was calculated from the use of 4 doses to determine the lethal dose (LD50); however, the selected doses provide 100% mortality or no infection (Supplementary Figure S1A). The mice were observed daily and euthanized using isoflurane anesthesia 15 days post-inoculation or at humane end points (paralysis, muscle abnormalities, and respiratory pattern alteration) according to survival analysis. For the short-term evaluation, mice were euthanized at 7 days using isoflurane anesthesia, with sample collection performed post-mortem (blood and brain). In the survival analysis, all mice were euthanized at the end of the experiment without the re-challenged virus. At 3 h before euthanasia, fluorescein isothiocyanate (FITC)-dextran, a nonabsorbable molecule with 4.4 kDa molecular mass (Sigma-Aldrich, St. Louis, MO, USA), was orally administered at a dose of at 12.5 mg in some mice (under isoflurane anesthesia), and FITC-dextran was detected in serum using a fluorospectrometer (Varioskan Flash Microplate Reader; Thermo Fisher Scientific, Waltham, MA, USA) to determine gut permeability defect. The detection of FITC-dextran in serum after oral administration indicates a gut permeability defect [27].

For the neurological screening, the SHIRPA (The SmithKline, Harwell, Imperial College, Royal Hospital, Phenotype Assessment) score was determined based on abnormal behavior (score 0–1), aggression (score 0–1), contact righting reflex (score 0–1), tremor (score 0–2), body tone (score 0–2), corneal reflex (score 0–2), negative geotaxis (score 0–4), pinna reflex (score 0–2), righting reflex (score 0–3), spontaneous activity (score 0–5), toe pinch (score 0–5), visual placing (score 0–5), and wire maneuver (score 0–5) [28]. Notably, the examples of abnormal behaviors are extreme fatigue, impaired motor control, and lack of coordination, while the instances of aggression include fighting, chasing, and injury. For the wire maneuver (a test of motor coordination and strength), a mouse was placed on a wire and observed for (i) the forepaw and hind paw grips and (ii) movement along the wire. There was no blind maneuver for scoring and euthanasia due to the extreme caution on rabies during the experiments. Mouse brain tissues and serum were stored at −80 °C before being used.

### 2.2. Mouse Sample Analysis

Blood urea nitrogen (BUN) and serum creatinine and were measured using the colorimetric method (QuantiChrom™ Urea and Creatinine Assay Kit, BioAssay System, Hayward, CA, USA), respectively, and alanine transaminase EnzyChrom Alanine Transaminase assay (EALT-100, BioAssay Systems, Hayward, CA, USA). Serum LPS (endotoxin) and cytokines (TNF-α, IL-6, and IL-10) were detected using the HEK-Blue LPS Detection Kit 2 (InvivoGen™, San Diego, CA, USA) and ELISA (Invitrogen, Carlsbad, CA, USA), respectively. Brain tissue was collected in RNAlater (Thermo Fisher Scientific), homogenized, and extracted using TRIzol™ Reagent before performing PCR, as mentioned above, using Rabies L protein (*RABL*) primers listed in Table 1. To monitor extraction efficiency, 10 ng of synthetic luciferase RNA transcript (Promega, Madison, WI, USA) was added to each sample before homogenization. Recovery of the spike-in RNA was verified by RT-qPCR. cDNA was synthesized using the High-Capacity cDNA Reverse Transcription Kit (Applied Biosystems, Thermo Fisher Scientific, Waltham, MA, USA), and quantitative PCR was performed using PowerUp™ SYBR™ Green Master Mix (Applied Biosystems, Thermo Fisher Scientific, Waltham, MA, USA) on a QuantStudio 6 Flex Real-Time PCR System (Applied Biosystems, Thermo Fisher Scientific, Waltham, MA, USA). The melting and standard curves are demonstrated in Supplementary Figure S1B. Primers targeting the rabies virus L gene were validated by 10-fold serial dilutions of plasmid standards (10^8^–10^2^ copies). The standard curve showed an efficiency of 64.5% and R^2^ = 0.989, with a single melt curve peak confirming specificity. The limit of detection (LOD) was determined to be approximately 100 copies per reaction. Viral copy numbers in brain samples were calculated from the standard curve and normalized to input brain tissue weight (mg).

For blood–brain barrier permeability analysis, the Evans blue dye (EB) assay was used following a published protocol [29]. Briefly, 1% EB (Sigma-Aldrich, St. Louis, MO, USA) at 2 mL/kg in 0.9% sodium chloride was administered via the tail vein at 30 min before sacrifice, and at sacrifice, phosphate-buffer solution (PBS) was perfused through the left ventricle until the blue color in blood was eliminated, and brains were weighed and snap-frozen in liquid nitrogen. Then, the brains were homogenized in formamide (Sigma-Aldrich, St. Louis, MO, USA) in a ratio of brain/formamide at 0.4 mg: 1 mL at 55 °C for 18 h before centrifugation, and EB in the supernatant was measured with an absorbance of 620 nm in comparison with the EB standard curve for the quantitative values. For viral abundance in the brain, the copy number of the virus was measured through the detection of the gene for L protein (a large multi-functional protein using an RNA-dependent RNA polymerase) with the PCR protocol, as mentioned above.

### 2.3. Fecal Microbiome Analysis

Mouse feces were collected from the mice in different cages (one mouse per cage) to avoid impacts of coprophagy (a habit where a mouse consumes feces from other mice in the same cage) before performing the previously described procedure [30,31]. Notably, additional nesting materials (cotton squares) were provided during the 7 days of the single housing. Briefly, 0.3 g of feces per mouse was used for the metagenomic DNA extractions using DNeasy Kit (Qiagen GmbH, Hilden, Germany), and the DNA quality was assessed by NanoDrop OneC Spectrophotometer (Thermo Fisher Scientific, Wilmington, DE, USA). Then, the universal prokaryotic primers 515F (5′-GTGCCAGCMGCCGCGGTAA-3′) and 806R (5′-GGACTACHVGGGTWTCTAAT-3′) with the appended 5′ Illumina adapter and 3′ Golay barcode sequences were used for 16S rRNA gene V4 library construction using the Mothur method. For 16S rRNA library sequencing, the prokaryotic 16S rRNA gene was performed using the Native barcoding kit (Oxford Nanopore Technologies, Oxford, UK). The targeted full-length 16S rRNA PCR protocol was carried out using the following cycling conditions: 95 °C for 1 min and 40 cycles of 95 °C for 20 s, 55 °C for 30 s, and 65 °C for 2 min, followed by 65 °C for 5 min. The 16S rRNA amplicons were purified by AMPure XP magnetic beads and ligated with different sequencing of Native barcode. The DNA libraries with different barcodes (approximately 1500 bp) were purified using AMPure XP beads (Beckman Coulter, Brea, CA, USA). The quality and quantity of DNA libraries were evaluated using DeNovix QFX Fluorometer (DeNovix, Wilmington, DE, USA) and QIAxcel Advanced (Qiagen, Hilden, Germany), respectively. Sequencing was performed using the Oxford nonopore MinION Mk1C platform following the manufacturer’s protocol (Oxford Nanopore Technologies, Oxford, UK). The basecalling and adapter trimming of raw sequences were performed using Dorado program version 0.7.3. Sequencing reads were filtered with DADA2 pipeline (min length = 1000, max length = 1600). The high-quality reads were processed following the Emu algorithm with the Emu database [32], and Emu established microbial community profiles. Alpha diversity index (Chao1 richness, and Shannon) and beta diversity were computed using R software version 4.3.0. Pairwise comparison of alpha diversity (observed ASVs, Chao1, and Shannon) was calculated using Kruskal–Wallis test (*p* < 0.05). Permutational multivariate analysis of variance (PERMANOVA) was performed to evaluate the significant differences for beta diversity among groups at *p* < 0.05. Moreover, the Kruskal–Wallis sum-rank test was also used in LEfSe analysis to identify bacterial biomarkers that differed significantly in abundant taxon between sample groups. The microbiome data set is available in the NCBI SRA database under BioProject PRJNA1266406.

### 2.4. The In Vitro Experiments

To investigate the macrophage responses to rabies virus, BV2 (murine microglial cell line) (ATCC CRL-2467) and bone-marrow-derived macrophages (BMMs) were maintained in supplemented DMEM. Notably, BMMs were derived from mouse femurs as previously described [33,34]. Because macrophage activation using viral antigens (pathogenic antigens) alone might be different from when viral antigens are used with antigens from the brain cells (self-antigens), BV2 or BMMs at 2 × 10^4^ cells/well in supplemented DMEM were incubated in 5% CO_2_ at 37 °C for 24 h before being used. The viral preparation using BHK-21 cells (mentioned above) at 2 × 10^4^ copies with and without brain preparation from the control mice (used 6.25 mg of brain tissue) were also tested in BV2 cells and BMMs. After 24 h of activation, the cell viability of the stimulation was determined by MTT (3-(4,5-dimethylthiazol-2-yl)-2,5-diphenyl tetrazolium bromide) assay (Thermo Fisher Scientific) [35]. Briefly, the cells were incubated with 0.5 mg/mL of MTT solution for 2 h at 37 °C in the dark and humidified with 5% CO_2_, MTT was removed, and cells were diluted with dimethyl sulfoxide (DMSO) before measurement with a Varioskan Flash microplate reader (Thermo Fisher Scientific, Waltham, MA, USA) at an absorbance of optical density of 570 nm. Supernatant cytokines were measured by ELISA (Invitrogen), while the expression of several genes was evaluated by PCR using the protocol mentioned above.

For Western blot analysis, BMDMs from WT and cGAS-/- mice (3 × 10^6^ cells) were lysed in RIPA (Radio-Immuno-Precipitation Assay) Lysis and Extraction Buffer (Thermo Scientific, Waltham, MA, USA) supplemented with protease and phosphatase inhibitors (ab201111; Abcam, Cambridge, UK). Protein concentration was determined by bicinchoninic acid assay (Pierce^TM^ BCA Protein Assay Kits; Thermo Fisher Scientific, Waltham, MA, USA). Equal amounts of protein (20 µg) were mixed with 4x Laemmli buffer, boiled for 5 min, separated on 10% SDS–PAGE gels, and transferred to Immun-Blot^®^ PVDF membranes (Bio-Rad Laboratories, Hercules, CA, USA). Membranes were blocked in 5% BSA (bovine serum albumin) in TBST (Tris-buffered saline with 0.05% Tween 20) buffer for 1 h at room temperature and incubated overnight at 4 °C with primary antibodies against cGAS (D3O8O) Rabbit mAb #31659 (Cell Signaling Technology, Danvers, MA, USA) or β-actin (13E5) Rabbit mAb #4970 (Cell Signaling Technology, Danvers, MA, USA). After washing, membranes were incubated with Anti-rabbit IgG, HRP-linked Antibody #7074 (Cell Signaling Technology, Danvers, MA, USA) secondary antibodies. Bands were visualized using ImageQuant™ LAS 500 (GE-Healthcare Life Sciences, Marlborough, MA, USA).

The levels of cyclic guanosine monophosphate–adenosine monophosphate (2′3′-cGAMP) (the secondary messenger of cGAS) was analyzed with an ELISA (Cayman Chemical, Ann Arbor, MI, USA; Catalog No. 501700) with a detection range of 6.1 pg/mL to 100 ng/mL according to the manufacturer’s protocols [36,37,38]. Samples were diluted to ensure their concentrations fell within this validated range. Mitochondrial oxidative stress was assessed by evaluating mitochondrial superoxide production using MitoSOX™ Red (Thermo Fisher Scientific, Waltham, MA, USA), a specific indicator of mitochondrial superoxide following the manufacturer’s protocol with minor modifications [36]. Following stimulation, cells were washed with pre-warmed Hank’s Balanced Salt Solution (HBSS) and incubated with 500 nM MitoSOX™ Red in the dark at 37 °C for 30 min. After incubation, cells were fixed with cold methanol and the fluorescence intensity was measured using a Varioskan™ Flash microplate reader with excitation and emission at 396 nm and 610 nm, respectively. In parallel, mitochondrial enumeration was also evaluated by mitochondrial DNA (mtDNA) using a tissue genomic DNA extraction mini kit (Favorgen Biotech, Wembley, WA, Australia) and NanoDrop ND-100 (Thermo Fisher Scientific, Waltham, MA, USA), normalized by β2-microglobulin (*β2M*) as previously described [36].

For the cell energy analysis, the Oxygen Consumption Rate (OCR) was measured using a Seahorse XFp Cell Mito Stress Test Kit (Agilent, Santa Clara, CA, USA) with Seahorse XFp Analyzers (Agilent, Santa Clara, CA, USA) as described in a previous publication [39]. In brief, macrophages were seeded (5 × 10^4^ cells/well) and stimulated, as described above, in a Seahorse cell culture plate before being replaced by Seahorse media (DMEM supplemented with 10 mM glucose, 1 mM pyruvate, and 2 mM glutamine) (Agilent, Santa Clara, CA, USA; 103575-100) for 1 h at 37 °C in a CO_2_-free incubator before activation by different metabolic interference compounds, including 1.5 µM of oligomycin, 1 µM of carbonyl cyanide-4 (trifluoromethoxy) phenylhydrazone (FCCP), and 0.5 µM of rotenone/antimycin A according to the protocol. All data were analyzed using Seahorse Wave 2.6 software based on the following equations: basal respiration = OCR before oligomycin − OCR after rotenone/antimycin A, maximal respiration = OCR between FCCP and rotenone/antimycin A − OCR after rotenone/antimycin A, ATP-linked respiration = OCR between FCCP and rotenone/antimycin A − OCR before oligomycin, and respiratory reserve = OCR between FCCP and rotenone/antimycin A − OCR before oligomycin.

### 2.5. Statistical Analysis

The results are shown as mean ± S.E.M. All data were analyzed with GraphPad Prism 6. One-way analysis of variance with Tukey’s comparison test was used for the analysis of experiments with more than two groups, and the survival analysis was determined by the log-rank test. A *p*-value less than 0.05 was considered significant.

## 3. Results

### 3.1. Less Severe Brain Inflammation and Gut Dysbiosis in Rabies-Infected cGAS-/- Mice

**Figure 1:**
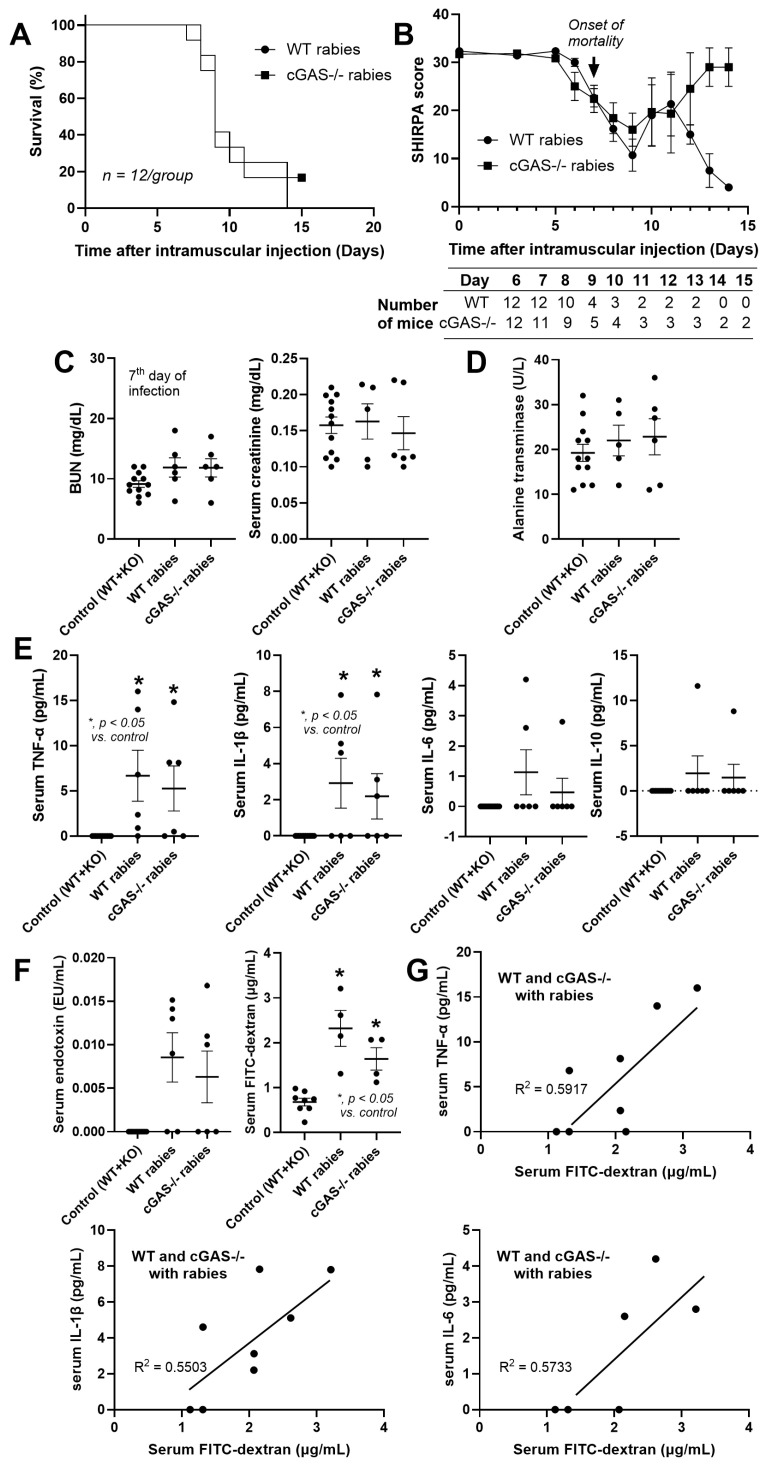
The characteristics of wild-type (WT) and cGAS-/- mice infected with rabies as indicated by survival analysis (*n* = 12/group) (**A**) and encephalopathy clinical score (SHIRPA score) (see method) (number of mice at 6 to 15 days after rabies injection is indicated in the table) (**B**). The characteristics on day 7 after rabies injection in control mice (WT combined with cGAS-/- mice) (*n* = 6/group, and the total number is 12/group), infected WT mice (*n* = 4–6/group), and infected cGAS-/- mice (*n* = 4–6/group), as indicated by renal functions using blood urea nitrogen (BUN) and serum creatinine (**C**), liver injury (serum alanine transaminase) (**D**), serum cytokines (TNF-α, IL-1β, IL-6, and IL-10) (**E**), gut permeability (serum endotoxin and FITC-dextran assay) (**F**), and the correlation between serum cytokines and gut permeability (FITC-dextran) (**G**). The control group is a combination of WT and cGAS-/- mice due to the similarity between these mice in the healthy condition [36].

With the CVS-11 rabies strain (a pathogenic virus for animal models) [40], there were 2 survivors out of 12 mice in the cGAS-/- group, whereas all infected WT mice died within 15 days after injection (Figure 1A). There was no statistically significant difference in the survival analysis with a *p*-value of 0.523 (log-rank test) (Figure 1A), and the power analysis value was 0.49 (MedCalc Software Ltd. Power estimator for Survival analysis (logrank test). https://www.medcalc.org/en/calc/power-survival-analysis.php (Version 23.4; accessed 12 October 2025). The survival curves started to drop 8 days post-infection (Figure 1A) with a decline in the SHIRPA score (Figure 1B), and the SHIRPA score at 9–10 days post-infection was affected by the score of survivors (Figure 1B, table). Nevertheless, there was no difference in the encephalopathy score between the mice. The score of the two survivors, however, decreased as early as 7 days post-injection and gradually recovered at 13 days post-injection (Figure 1B), indicating a successful infection with self-recovery in these two mice. At 7 days post-rabies injection, the data from the control WT and cGAS-/- groups were combined (WT + KO) because of the non-difference between groups in the controls, as previously mentioned [36]. There was no renal or liver injury from rabies in both mouse strains (Figure 1C,D), while serum TNF-α and IL-1β (but not IL-6 and IL-10) were similarly elevated (Figure 1E), supporting a previous publication [41]. Due to stress-induced leaky gut (the translocation of microbial molecules from the gut into the blood circulation) [42], serum endotoxin (LPS) and FITC-dextran assay (see method) were analyzed. As such, gut permeability defect (leaky gut) was detected by the FITC-dextran assay, but not endotoxemia, in the infected mice (similar in both mouse strains) (Figure 1F). Notably, the detection of non-gut absorbable FITC-dextran in the blood after oral administration indicates a leaky gut condition [43]. The rabies-induced leaky gut might partly be a cause of systemic inflammation, as indicated by the correlation between serum cytokines and the FITC-dextran assay with an r-square value in the range of 0.55–0.59 (55–59% of serum cytokines explained by the factor of leaky gut) (Figure 1G).

**Figure 2:**
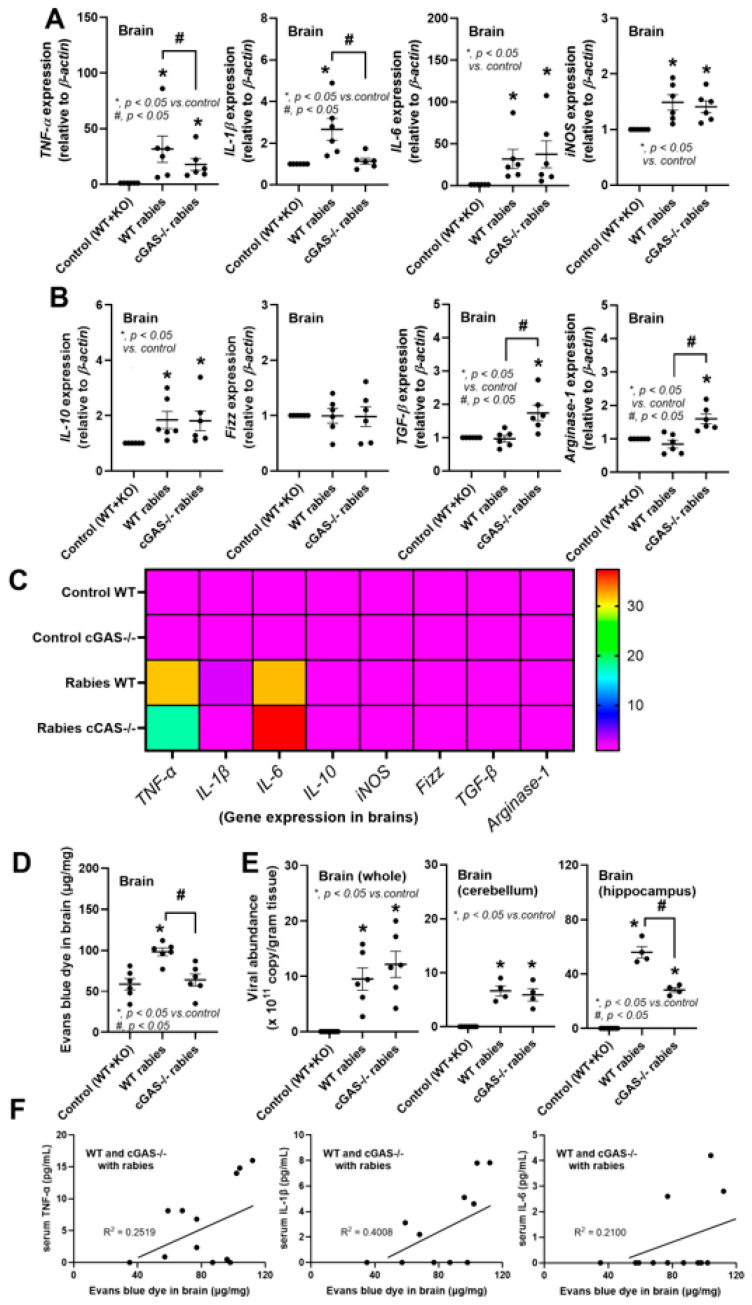
The characteristics of wild-type (WT) (*n* = 4–6/group) and cGAS-/- mice (*n* = 4–6/group) infected on day 7 after rabies injection or control mice (WT combined with cGAS-/- mice) (*n* = 6/group and the total number is 12), as indicated by gene expression in the brain tissue for proinflammatory genes (*TNF-α*, *IL-1β*, *IL-6*, and *iNOS*) (**A**), anti-inflammatory genes (*IL-10*, *Fizz*, *TGF-β*, and *Arginase-1*) (**B**) with the heat map presentation (**C**), blood–brain barrier defect (Evans blue dye assay) (**D**), the viral abundance in the whole brain, cerebellum, and hippocampus (**E**), and the correlation between serum cytokines and blood–brain barrier defect (Evans blue dye assay) (**F**). The control group is a combination of WT and cGAS-/- mice due to similarities between these mice while in a healthy state [36].

Then, brain inflammation was determined based on the expression of several genes. As such, the brains of rabies-infected WT mice demonstrated higher *TNF-α* and *IL-1β*, but not *IL-6* and *iNOS*, when compared with the infected cGAS-/- brains (Figure 2A). Meanwhile, the infected cGAS-/- brains demonstrated more prominent anti-inflammation, as indicated by *TGF-β* and *Arginase-1*, but not *IL-10* and *Fizz* (Figure 2B). With the heat map presentation, the inflammation in rabies-infected brains was mainly demonstrated more through the expression of *TNF-α* and *IL-6* than the other parameters, while also indicating the similarity of the control WT and cGAS-/- (Figure 2C). Additionally, the more prominent Evans blue dye color in the infected WT brains than in the cGAS-/- (Figure 2D) indicated a rabies-induced blood–brain barrier (BBB) defect [44,45]. The viral abundance in the whole brain and in the cerebellum was similar in both mouse strains, while there was more prominent viral abundance in the hippocampus of WT mice (Figure 2E). There was a low correlation (low r-square) between serum cytokines and BBB defect (Figure 2F). These data indicate that rabies infection in the brain is less likely to induce systemic inflammation, and we hypothesized that systemic inflammation (elevated serum TNF-α and IL-1β) in mice (Figure 1E) might partly be due to stress-induced gut dysbiosis.

Overall, cGAS deficiency was correlated with a lower level of rabies-induced brain inflammation and a similar level of systemic inflammation compared with infected WT mice. Notably, data of the survivors did not skew day 7 parameters because these two mice were euthanized at the end of the survival experiment (15 days) without sample evaluation following the animal study protocol. Despite the non-difference in the survival analysis with low statistical power that might have led to a type II error (failing to reject the null hypothesis when it should be rejected), there was a trend that aligns with other findings of reduced severity in rabies-infected cGAS-/- mice when compared to infected WT mice.

**Figure 3:**
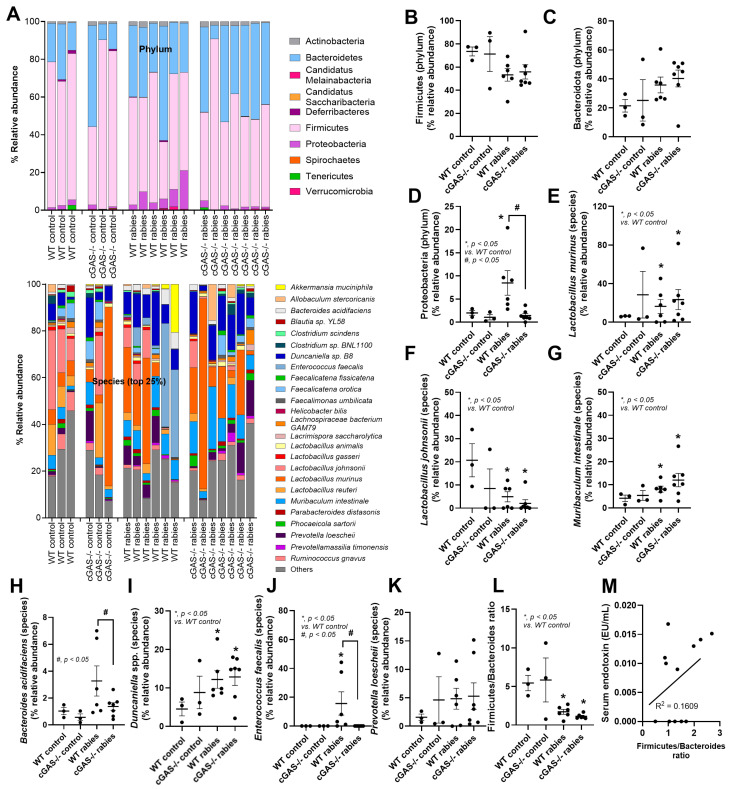
The characteristics of wild-type (WT) and cGAS-/- mice injected with vehicle control or rabies virus at 7 days post-injection, as indicated by fecal bacterial abundance in the phylum and species (top 20% highest abundance) levels (**A**), and the graph presentation of the selected fecal microbiome analysis in the phylum and species levels (**B**–**K**), Firmicutes/Bacteroides (F/B) ratio (**L**), and the correlation between serum endotoxin and F/B ratio (**M**) (*n* = 3/group for WT and cGAS-/- control, *n* = 6 for WT with rabies, and *n* = 7 for cGAS-/- with rabies).

To investigate stress-induced dysbiosis, a fecal microbiome analysis was performed. Overall, the approximate saturation of microbial richness of all samples was 20,685 sequencing depths, as estimated by the rarefaction curves. The plateau curve in rarefaction was observed when approximately 15,000 sequencing depths were reached. Non-Metric Multidimensional Scaling (NMDS), based on Bray–Curtis, showed that microbiota communities were distinct among the groups (PERMANOVA test; *p* = 0.000999). This data indicates that microbiota communities were different among the groups. Additionally, the fecal microbiome analysis (the abundance of fecal bacteria) of mice after 7 days in the control or in mice injected with rabies was demonstrated at the phylum and species levels (Figure 3A), while other data are shown in Supplementary Figure S2. Some microbiome analyses are further presented (Figure 3B–K). As such, the phylum Proteobacteria (including several pathogenic Gram-negative bacteria), but not Firmicutes and Bacteroidota, in the rabies-infected WT mice was higher than in other groups (Figure 3B–D). Meanwhile, Proteobacteria in the rabies-infected cGAS-/- mice was not different from the control (Figure 3B–D). With the selected species-level comparison of fecal microbiome (Figure 3E–K), there was a similar alteration between WT and cGAS-/- mice with rabies infection, except for the higher abundance of *Bacteroides acidifaciens* and *Enterococcus faecalis* in the rabies-infected WT mice (Figure 3H,J). The Firmicutes/Bacteroides (F/B) ratio (one of the dysbiosis parameters [46]) in the rabies-infected mice was similar between mouse strains and lower than the control (Figure 3L). However, the correlation (r-square) between endotoxemia and the F/B ratio was low, indicating the limitation of the F/B ratio as a representative of the dysbiosis parameter [46].

**Figure 4:**
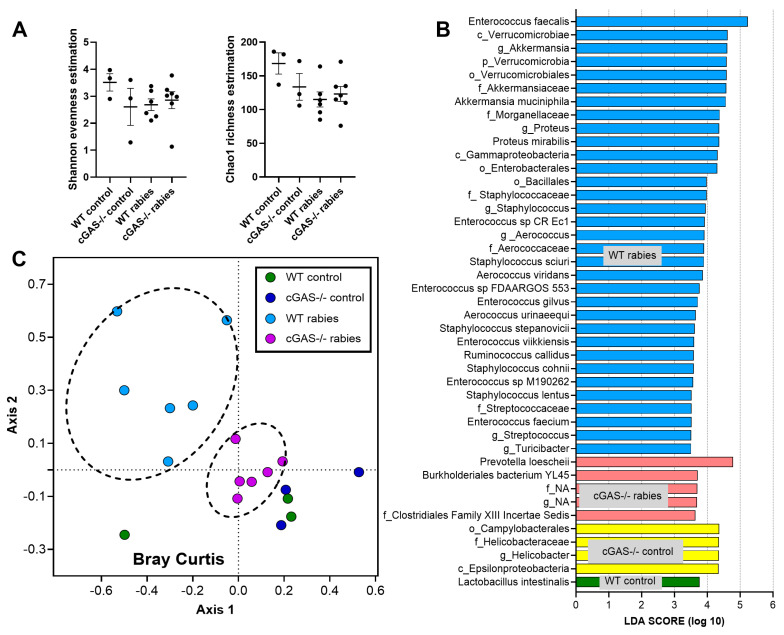
The characteristics of wild-type (WT) and cGAS-/- mice injected with vehicle control or rabies virus at 7 days post-injection, as indicated by the alpha diversity indexes obtained using Shannon evenness estimation (the distribution of individuals among species) and Chao-1 richness estimation (the number of different species) (**A**), the Linear discriminant analysis Effect Size (LEfSe) (a combination of statistical tests to identify the representative bacteria that significantly differ among groups) (**B**), and the beta diversity using the Bray–Curtis dissimilarity index (the demonstration of differences among samples through distance from axis) (**C**) (*n* = 3/group for WT and cGAS-/- control, *n* = 6 for WT with rabies, and *n* = 7 for cGAS-/- with rabies).

The alpha diversity indexes (Shannon and Chao-1 estimation) were not different among groups (rabies infection and control) (Figure 4A). Meanwhile, both the Linear discriminant analysis Effect Size (LEfSe) (the representative bacteria for each group) and the beta diversity (the Bray–Curtis dissimilarity index) indicated differences (Figure 4B,C). The LEfSe showed that several bacteria, especially *Enterococcus faecalis* and *Verrucomicrobiae*, were representatives of rabies-infected WT mice, while *Prevotella* sp. and *Burkholderia* represented rabies-infected cGAS-/- mice (Figure 4B). Meanwhile, *Campylobacter* spp. and *Lactobacillus* sp. were representative of the cGAS-/- and WT control groups, respectively (Figure 4B). The Bray–Curtis dissimilarity index (the beta diversity indicating the differences between groups through the 2-dimensional distances from the axis) indicated a similarity between the WT and cGAS-/- mice with rabies infection (no obvious difference between control groups of both mouse strains) (Figure 4C). Notably, the gut permeability defect (indicated by FITC-dextran) (Figure 1F, right side), together with fecal dysbiosis, supported the gut–brain axis in rabies infection [47,48].

### 3.2. The Less Prominent Macrophage Responses in cGAS-/- than in WT Cells Implied Rabies-Induced Mitochondrial Damage

**Figure 5:**
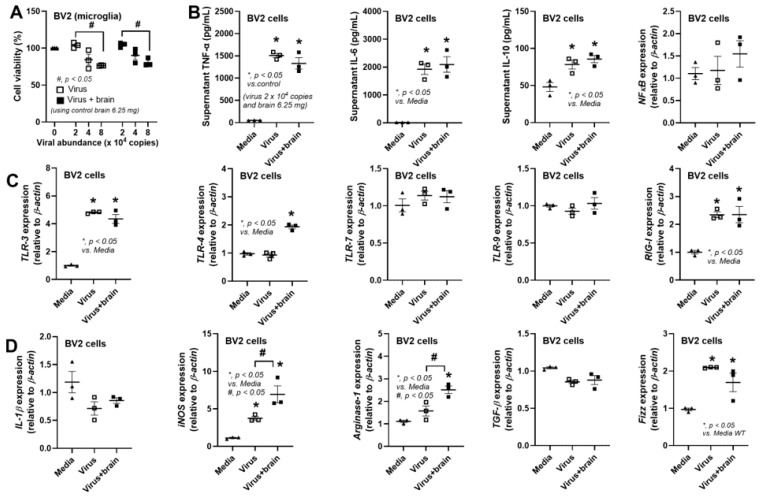
The characteristics of BV2 cells (a microglia cell line) after 24 h of activation by rabies virus (2 × 10^4^ copies) with and without brain preparation from the WT control mice (6.25 mg), as indicated by cell viability (**A**), proinflammatory markers using supernatant cytokines (TNF-α, IL-6, IL-10, and *NF-κB* expression) (**B**), genes of pattern recognition receptors (*TLR3*, *TLR-4*, *TLR-7*, *TLR-9*, and *RIG-I*) (**C**), genes of proinflammatory markers (*IL-1β* and *iNOS*), and anti-inflammatory markers (*Arginase-1*, *TGF-β*, and *Fizz*) (**D**).

The rabies virus with and without brain preparation from the control mice was tested in BV2 cells (a microglia cell line) to explore the differences between activation by the virus alone and by the virus with self-antigens. Due to reduced cell viability from the rabies virus at 8 × 10^4^ copies (a toxic level) (Figure 5A), the virus at 2 × 10^4^ copies with a multiplicity of infection (MOI) value of 1.0, with and without brain preparation (6.25 mg brain), was further used. Either the virus alone or the virus + brain similarly elevated supernatant cytokines (TNF-α, IL-6, and IL-10) without an impact on *NFκB* expression (Figure 5B). Meanwhile, the virus alone upregulated *TLR-3* and *RIG-1*, while the virus + brain elevated *TLR-3*, *TLR-4*, and *RIG-1* (Figure 5C). Perhaps these RNA recognition receptors (TLR-3, RIG-1, and MDA-5) recognized the virus, while TLR-4 detects antigens from brain tissue [49]. Notably, *TLR-9* was measured with the aim of exploring the possible recognition of the host DNA [50]. For macrophage polarization of the microglia (BV2), both the virus and virus + brain elevated *iNOS*, *Arginase-1*, and *Fizz*, but the virus + brain induced higher levels of *iNOS* and *Arginase-1* (Figure 5D). These data support the impact of damage-associated molecular patterns (DAMPs) from brain tissue during innate immune responses against rabies [3].

**Figure 6:**
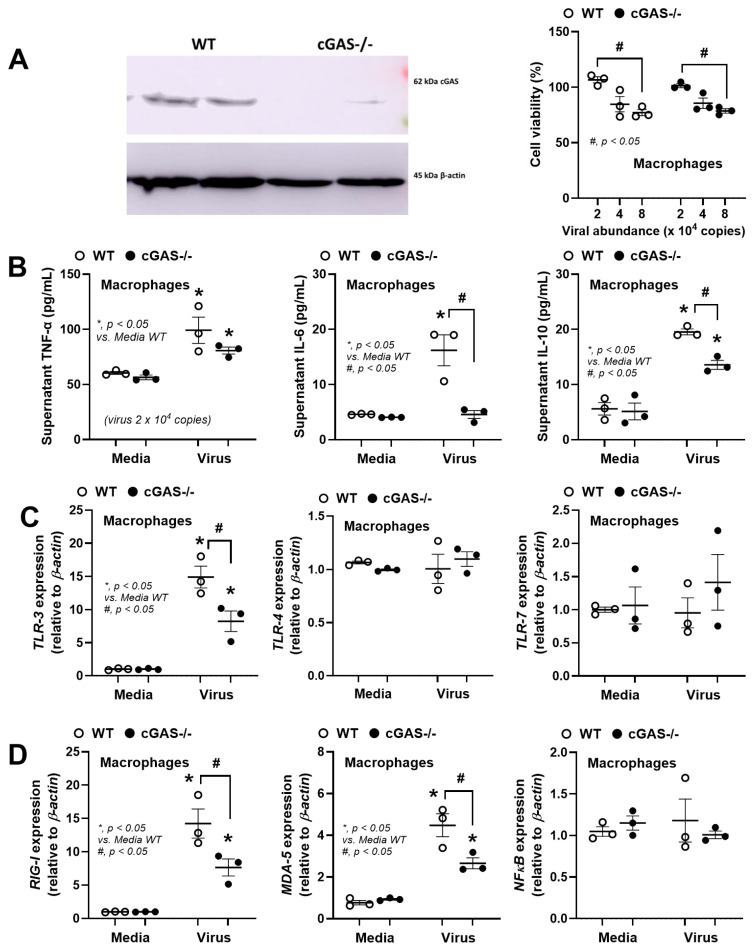
The characteristics of bone-marrow-derived macrophages from wild-type (WT) and cGAS-/- mice after 24 h activation by rabies virus (2 × 10^4^ copies) with and without brain preparation from the WT control mice (6.25 mg), as indicated by the representative Western blot analysis and cell viability (**A**), supernatant proinflammatory cytokines (TNF-α, IL-6, and IL-10) (**B**), genes of pattern recognition receptors (*TLR3*, *TLR-4*, *TLR-7*, *TLR-9*, and *RIG-I*) (**C**), and genes of interferon (IFN) and cytokine production (*RIG-1*, *MDA-5*, and *NF-κB*) (**D**).

**Figure 7:**
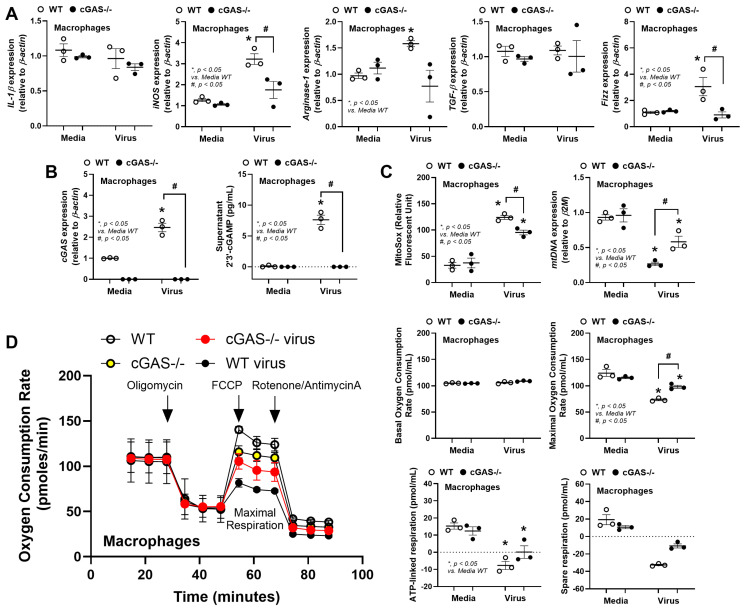
The characteristics of bone-marrow-derived macrophages from wild-type (WT) and cGAS-/- mice after 24 h activation by rabies virus (2 × 10^4^ copies) with and without brain preparation from the WT control mice (6.25 mg), as indicated by genes of M1 macrophage polarization (*IL-1β* and *iNOS*) and M2 macrophage polarization (*Arginase-1*, *TGF-β*, and *Fizz*) (**A**), *cGAS* expression and supernatant 2’3’-cGAMP (a cGAS inducer) (**B**), markers of mitochondrial injury using MitoSox and expression of mitochondrial DNA (mtDNA) (**C**), and extracellular flux analysis using oxygen consumption rate (OCR) with several parameters (**D**).

After that, experiments to explore the possible different responses between WT and cGAS-/- macrophages were performed using bone-marrow-derived macrophages (BMMs). As such, the deficiency of cGAS protein was demonstrated by Western Blot analysis (Figure 6A, left side; uncropped blots are shown in Supplementary Material), and the virus at 2 x 10^4^ copies was used due to there being no change in cell viability (Figure 6A, right side). There were more prominent supernatant cytokines (TNF-α, IL-6, and IL-10) in the WT BMMs than cGAS-/- cells (Figure 6B). The rabies virus more prominently upregulated *TLR-3*, *RIG-1*, and *MDA-5*, but not *TLR-4*, *TLR-7*, and *NFκB*, in the WT macrophages when compared with cGAS-/- cells (Figure 6C,D). For macrophage polarization genes, rabies upregulated *iNOS*, *Arginase-1*, and *Fizz* only in the WT cells, and not in cGAS-/- cells (Figure 7A). The lower responses to rabies in cGAS-/- cells compared to WT cells implied the activation of cytosolic DNA, possibly from the presence of mitochondrial DNA (rabies-induced mitochondrial injury) [15,51,52] to the cGAS receptors. Then, several mitochondrial injuries and cGAS parameters were evaluated. Accordingly, the rabies virus upregulated the *cGAS* gene, increased supernatant cGAMP (a secondary messenger of cGAS), elevated mitochondrial oxidative stress (MitoSox), and reduced the amount of mitochondria (low mitochondrial DNA) more prominently in WT cells when compared to cGAS-/- cells (Figure 7B,C). These data support the impacts of rabies virus in mitochindrial injury. Notably, the elevated MitoSox in cGAS-/- macrophages, despite the absence of cGAS and cGAMP, indicates non-cGAS-mediated mitochondrial injury. Additionally, a more prominent Reduction In Mitochondrial function in rabies-activated WT macrophages than cGAS-/- cells was also indicated by extracellular flux analysis (Figure 7D).

As such, rabies induced lower maximal respiration (a capacity to increase ATP production) and ATP-linked respiration (oxygen consumption for oxidative phosphorylation-related ATP production) without changes in basal respiration (oxygen consumption rate at cell rest) and spared respiratory capacity (an ability to respond to increased energy demand) (Figure 7D). The maximal respiration of rabies-activated WT macrophages was lower than the activated cGAS-/- cells (Figure 7D), implying the better capacity of cGAS-/- macrophages to increase ATP, possibly due to the less severe responses.

## 4. Discussion

The rabies-induced inflammation in cGAS-/- mice was less severe than in WT mice partly because of stress-induced gut dysbiosis due to the exposure of mitochondrial DNA in the cytosol that can activate cGAS (a cytosol DNA receptor). The influence of innate immunity in rabies is interesting.

### 4.1. Less Severe Inflammation in Rabies-Infected cGAS-/- Mice: Roles of Rabies-Induced Mitochondrial Injury

While the survival analysis was not different between the WT and cGAS-/- mice with rabies, surviving mice were found only in the cGAS-/- group. While systemic inflammation (serum cytokines) was not different between mouse strains, the viral burdens and blood–brain barrier (BBB) defect were more prominent in the infected WT mice. These data indicate a possible less severe rabies infection in cGAS-/- mice than in WT mice. The lower expression of *TNF-α* and *IL-1β* (proinflammatory markers) with higher *TGF-β* and *Arginase-1* (anti-inflammatory genes) in rabies-infected cGAS-/- brains compared with those in the WT brains was demonstrated. These data imply possible cGAS-mediated inflammation in brain tissue. Notably, the microglia (macrophages in the brain) and astrocytes (macroglia) are major sources of TNF-α, TGF-β, and IL-1β in the brain [53], while the source of Arginase-1 is mainly microglia [54]. The alteration in these cytokines highlights the roles of innate immune cells in rabies. While TNF-α and IL-1β activate virucidal activities, both cytokines also induce inflammation, damage the BBB, and might worsen rabies symptoms [5,55]. On the other hand, TGF-β and Arginase-1 (markers of M2 anti-inflammatory macrophage polarization) [56] suppress immune responses, promote tissue repair (TGF-β), and down-regulate nitric oxide synthesis (Arginase-1), resulting in less severe inflammation [39,57]. The lower proinflammatory and higher anti-inflammatory molecules in rabies-infected cGAS-/- brains compared to the WT led to less severe inflammation and BBB damage but with intact virucidal activity. Despite the similar viral burdens in the whole brain and in the cerebellum between mouse strains, the burdens in the hippocampus of the infected cGAS-/- mice were lower than in the WT mice. Hence, the reduced inflammation due to the lack of cGAS receptors might decrease viral burdens. While the inflammatory response is crucial for fighting infection, an exaggerated inflammatory state can be hijacked by viruses or cause collateral damage that impairs effective viral clearance and increases the viral burdens in the brains [58,59]. Because the cGAS receptor is involved in rabies neuroinflammation but not responsible for virucidal activities, cGAS blockage might be an interesting strategy to control brain damage in rabies.

However, in vitro experiments using rabies on a microglia cell line (BV2 cells) indicated that rabies increased supernatant inflammatory cytokines, especially TNF-α, and upregulated only *iNOS* and *Fizz*, possibly through *TLR-3*, *RIG-1*, and *MDA-5* [60]. Meanwhile, viruses with brain preparations facilitated inflammatory responses through *TLR-4* and *iNOS*. Additionally, the elevated *TGF-β* and *Arginase-1* (M2 polarization) in mouse brains might be due to several factors, for example, Th2 [61] from rabies activation [62]. Nevertheless, these data support the differences between in vitro and in vivo experiments. Moreover, the elevation in *TLR-3*, *RIG-1*, and *MDA-5*, but not *TLR-7*, in rabies-activated microglia may depend on the duration and dose of the viruses. While TLR-3, RIG-1, and MDA-5 primarily recognize double-stranded RNA (dsRNA), their activation by the rabies virus (a single-stranded RNA virus) is possibly due to the virus’s replication process with specific RNA intermediates, for example, 5′ copy-back defective interfering (5′cb DI) RNAs that mimic dsRNA structures and trigger these receptors [63,64,65]. Despite the selected dose of CVS-11 virus with 24 h of incubation not upregulating *TLR-7*, the elevation in TLR-7 (a receptor for ssRNA) in BV2 cells after 72 h of incubation with rabies has been reported [66].

For in vitro macrophage polarization, the upregulation of both *iNOS* and *Arginase-1* indicates an unclear categorization of macrophage polarization but possibly a balance of immunological processes (Arginase-1 interferes with iNOS) [56]. Rabies activation in WT macrophages did not promote a pure M1 phenotype with broad and hyperactive functions but induced a dysregulated macrophage state. Although pure canonical M1 macrophage polarization promotes potent virucidal activity, rabies shifts the polarization towards some characteristics of the M2-like polarization, an anti-inflammatory state, possibly as a part of viral evasion [67]. Hence, rabies did not uniquely reprogram macrophages in either M1 or M2, which indicates immunomodulatory characteristics [67] that might partly reduce the potency of virucidal activity. The impact of rabies on macrophages might partly be due to rabies-induced mitochondrial injury [7]. As such, mitochondria are prokaryotes with circular DNA that symbiotically stay inside eukaryotic cells as an organelle for energy production. Severe macrophage activation (such as in sepsis) damages mitochondria partly through oxidative stress, resulting in the releasing of free mitochondrial DNA into the cytosol that activates cGAS [38]. In rabies, the viral phosphoprotein (P) directly damages mitochondria through interaction with complex I, resulting in oxidative stress production in infected cells (neurons and macrophages) [15,51,52]. Indeed, the activation of cGAS by the rabies virus was supported by the upregulated cGAS and production of cGAMP in WT macrophages. Despite the less severe responses in cGAS-/- macrophages than in WT cells, rabies still significantly injured cGAS-/- macrophages (mitochondrial oxidative stress, mtDNA, and extracellular flux analysis). These data imply that the rabies virus can damage mitochondria, and cGAS blockage alone is not enough to neutralize rabies-induced neuroinflammation. Additional studies within this discipline are of great interest.

### 4.2. Stress-Induced Dysbiosis, an Interesting Gut–Brain Axis in Rabies

Similarly, elevated serum cytokines in rabies-infected WT and cGAS-/- mice were presented despite the neurotrophic nature of the virus. The correlation between the FITC-dextran assay (a leaky gut parameter) and serum cytokines supported the gut–brain axis correlation. The rabies-induced gut–brain axis might be due to (i) the virus in the gut vagal nerve [68,69] or (ii) stress-induced gut dysbiosis (the selective growth of some bacterial species due to different responses against the host’s neurotransmitters) [21,22,23]. Both the rabies virus (or immunization) [70,71] and infection-induced stress elevate serum cytokines, perhaps through the sympathetic pathway [72,73]. Interestingly, stress can induce gut dysbiosis that is severe enough to generate intestinal permeability defects (leaky gut) and endotoxemia [42]. Hence, it seems that the stress from rabies infection alone, regardless of rabies transmission through the vagal nerve, might facilitate gut dysbiosis. Notably, during leaky gut, lipopolysaccharide (LPS), a major component of Gram-negative bacteria (the most abundant organisms in the gut), can translocate from the gut into the blood circulation, causing endotoxemia and systemic inflammation [28,74]. Despite no endotoxemia here (possibly due to the too short duration of stress), the FITC-dextran assay supported rabies-induced leaky gut that might be partly derived from gut dysbiosis.

Indeed, fecal dysbiosis was demonstrated in both mouse strains compared to non-rabies infection, as indicated by (i) FITC-dextran and (ii) a reduced Firmicutes/Bacteroides ratio in the infected mice. The Bray–Curtis (the differences demonstrated by distances to the axis) and LEfSe analyses (the representative bacteria for the group) demonstrated differences between WT and cGAS-/- mice after infection. Interestingly, Proteobacteria (phylum), *Bacteroides* sp., and *Enterococcus* sp. contents in rabies-infected WT mice were higher than in the infected cGAS-/- mice (Figure 3D,H,J). As such, Proteobacteria, or Pseudomonadota, is a major phylum of Gram-negative bacteria that includes several pathogenic organisms, for example, *Escherichia coli*, *Salmonella* spp., and *Klebsiella* spp. [75]. The elevated Proteobacteria content in fecal microbiome analysis is a possible microbial signature of dysbiosis in several diseases [75]. Meanwhile, *B. acidifaciens* are Gram-negative anaerobes in the phylum Bacteroides with several protective properties in several diseases (obesity, liver diseases, and diabetes) [76]. However, Bacteroides bacteremia is reported in some immunocompromised hosts [77]. On the other hand, *E. faecalis* are facultative anaerobes in the phylum Firmicutes that can be beneficial or harmful to the host [78], and some specific strains of *E. faecalis* are used as probiotics [79]. Although there was an elevation in Proteobacteria (a dysbiosis parameter) in rabies-infected WT mice, an increase in *B. acidifaciens* and *E. faecalis* (the possible beneficial bacteria) might neutralize the negative impact of Proteobacteria, leading to similar systemic inflammation to the infected cGAS-/- mice. Because fecal microbiome analysis was evaluated at 7 days post-infection (the onset of encephalopathy according to the SHIRPA score), fecal microbiome characteristics at this time point should be a result of rabies infection but not a cause of disease severity. The time point evaluation of fecal microbiome analysis and the evaluation of the disease severity after the attenuation of fecal dysbiosis and leaky gut are needed to determine the cause–effect of rabies and the intestinal impacts.

Notably, the influence of immune response on fecal microbiome was previously mentioned in liposomal clodronate-induced macrophage depletion through the accumulation of bacteria that need macrophages to control their population [80]. Differences in immune responses between WT and cGAS-/- mice after rabies infection might affect fecal dysbiosis. Despite the similar baseline characteristics between WT and cGAS-/- mice, cGAS-/- macrophages demonstrate less prominent functions during stress and infection [81] from the absence of cytosolic DNA signaling that might lead to an increase in some populations of bacteria. Hence, our data support a gut–brain axis in rabies infection. However, the importance and influence of gut microbiota alteration in rabies needs more investigations. The lower inflammatory responses to rabies in cGAS-/- mice compared to WT mice may be due to the interference of rabies on mitochondria and the lack of response against mitochondrial DNA in the cytosol, which would result in lower activity of cGAS-/- cells.

### 4.3. Clinical Aspects of the Study

Although rabies is currently a lethal disease with only symptomatic treatment with 100% fatalities in symptomatic cases [82,83], attenuation of the disease severity in some aspects might be the beginning of a novel management technique, at least to reduce suffering and distress in patients. Due to the possible impacts of the cGAS receptor in rabies as mentioned here and the availability of cGAS inhibitors, including some small molecules, natural products (flavonoids), and antimalarial drugs (hydroxychloroquine and quinacrine) [84,85,86], the use of these compounds might be beneficial in treating rabies. Moreover, the possible importance of mitochondria in the pathogenesis of rabies implies the possibility to alter the natural history of rabies infection by improving mitochondrial functions using several compounds (coenzyme Q10, antioxidants, and mitoquinone) [87,88,89]). Hence, rapid administration of cGAS inhibitors and/or mitochondrial strengthening right after the incidence of rabies infection (animal bites) might interfere with rabies pathogenesis that might affect the natural history of the disease. Additional studies are needed.

### 4.4. Study Limitations

First, the CVS-11 laboratory strain but not the street virus (the strains isolated from naturally infected animals) was used. Street viruses are more virulent and cause more diffuse neuron distribution with a longer incubation period than the CVS-11 strain [90]. More studies using street viral strains are warranted. Second, only male mice were used. Despite limited reports on the comparison of rabies responses between males and females, post-menopausal women demonstrate a weaker response to rabies vaccines [91]. This data implies a possible gender difference in rabies natural response that might be interesting to test in a larger number of animals. Third, there was a limited number of mice overall in the study and in several experiments. Several conclusions were derived from a small sample size, and the conclusions might be different with an adequate number of mice. For example, the FITC-dextran assay was performed only on five mice per group, and the survival analysis was underpowered. Additionally, the rabies-induced gut permeability defect and the lack of differences found in the survival analysis of the WT and cGAS-deficient mice with rabies were preliminary. Thus, more studies are warranted. Fourth, our study lacks the condition that mimics human rabies, especially the post-exposure immune responses. Hence, the role of cGAS enhancers to elevate immune responses during post-exposure vaccination is intrinsically worth exploring. Fifth, the source of cytosolic DNA might also be due to self-DNA from the host cells because rabies can induce cell apoptosis, oxidative stress, and DNA damage (the induction of the translocation of self-DNA from the nucleus to the cytosol) [92]. Thus, self-DNA might be another cGAS inducer during rabies infection. Sixth, the evidence for mitochondrial injury remains indirect with only subtle changes. As such, mitochondrial injury, as evaluated by the MitoSox assay in rabies-treated macrophages, was only approximately 1.5-fold higher than the control. Supplementary studies to illuminate this narrative would be of interest.

## 5. Conclusions

The inflammation in the brains of cGAS-/- mice was less than in WT mice, without an alteration in the virucidal effect. These data imply that there were impacts of rabies-induced mitochondrial injury, and interference with cGAS might be beneficial to control innate immune responses against rabies, which might limit the neurological damage caused by rabies. On the other hand, stress-induced gut dysbiosis was observed in rabies and might be another interesting topic for in-depth exploration. More studies are warranted.

## Figures and Tables

**Table 1:** List of primers used in the study.

Name	Forward	Reverse	Amplicon Size (bp)
Rabies L protein (*RABL*) Gene ID: 1489857	5′-GAGAGCCGTCTCTTAGAGGA-3′	5′-GCGCGACACCTTCTTGTTAG-3′	470
Toll-like receptor 3 (*TLR-3*) Gene ID: 142980	5′-GTCTTCTGCACGAACCTGACAG-3′	5′-TGGAGGTTCTCCAGTTGGACCC-3′	164
Toll-like receptor 4 (*TLR-4*) Gene ID: 21898	5′-GGCAGCAGGTGGAATTGTAT-3′	5′-AGGCCCCAGAGTTTTGTTCT-3′	198
Toll-like receptor 7 (*TLR-7*) Gene ID: 170743	5′-GTGATGCTGTGTGGTTTGTCTGG-3′	5′-CCTTTGTGTGCTCCTGGACCTA-3′	100
Toll-like receptor 9 (*TLR-9*) Gene ID: 81897	5′-GCTGTCAATGGCTCTCAGTTCC-3′	5′-CCTGCAACTGTGGTAGCTCACT-3′	115
Retinoic acid-inducible gene I (*RIG-I*) Gene ID: 230073	5′-CCACCTACATCCTCAGCTATATGA-3′	5′-TGGGCCCTTGTTGTTCTTCT-3′	86
Melanoma differentiation-associated protein (*MDA-5*) Gene ID: 71586	5′-GCCTGGAACGTAGACGACAT-3′	5′-TGGTTGGGCCACTTCCATTT-3′	249
Cyclic GMP–AMP synthase (*cGAS*) Gene ID: 214763	5′-ATGTGAAGATTTCGCTCCTAATGA-3′	5’-GAAATGACTCAGCGGATTTCCT-3’	145
Inducible nitric oxide synthase (*iNOS*); Gene ID: 18126	5′-ACCCACATCTGGCAGAATGAG-3′	5′-AGCCATGACCTTTCGCATTAG-3′	111
Interleukin-1β (*IL-1β*) Gene ID: 16176	5′-GAAATGCCACCTTTTGACAGTG-3′	5′-TGGATGCTCTCATCAGGACAG-3′	116
Tumor necrosis factor α (*TNF-α*) Gene ID: 21926	5′-CCTCACACTCAGATCATCTTCTC-3′	5′-AGATCCATGCCGTTGGCCAG-3′	135
Interleukin-6 (*IL-6*) Gene ID: 16193	5′-TACCACTTCACAAGTCGGAGGC-3′	5′-CTGCAAGTGCA TCA TCGTTGTTC-3′	116
Interleukin-10 (*IL-10*) Gene ID: 16153	5′-GCTCTTACTGACTGGCATGAG-3′	5′-CGCAGCTCTAGGAGCATGTG-3′	105
Arginase-1 (*Arg-1*) Gene ID: 11846	5′-CTTGGCTTGCTTCGGAACTC-3′	5′-GGAGAAGGCGTTTGCTTAGTT-3′	146
Resistin-like molecule-α1 (*FIZZ-1*) Gene ID: 57262	5′-GCCAGGTCCTGGAACCTTTC-3′	5′-GGAGCAGGGAGATGCAGATGA-3′	102
Transforming growth factor-β (*TGF-β*) Gene ID: 21813	5′-CAGAGCTGCGCTTGCAGAG-3′	5′-GTCAGCAGCCGGTTACCAAG-3′	106
Nuclear factor kappa B (*NFκB*) Gene ID: 18033	5′-CTTCCTCAGCCATGGTACCTCT-3′	5′-CAAGTCTTCATCAGCATCAAACTG-3′	167
*β-actin* Gene ID: 11461	5′-CGGTTCCGATGCCCTGAGGCTCTT-3′	5′-CGTCACACTTCATGATGGAATTGA-3′	100
Mitochondrial DNA (*mtDNA*) Gene ID: PV231059.1	5′-CGTACACCCTCTAACCTAGAGAAGG-3′	5′-GGTTTTAAGTCTTACGCAATTTCC-3′	70
β2-microglobulin (*β2M*) Gene ID: NM_009735.3	5′-TTCTGGTGCTTGTCTCACTGA-3′	5′-CAGTATGTTCGGCTTCCCATTC-3′	104

## Data Availability

Raw microbiome sequencing data from this study have been deposited in the NCBI SRA under BioProject accession PRJNA1266406. All other data that support the findings of this study are available in the article and its Supplementary Materials.

## Supplementary Materials

The following supporting information can be downloaded at: https://www.mdpi.com/article/10.3390/biology14111583/s1, Figure S1. The determination of LD50 (Lethal Dose 50%) of CVS-11 rabies strain using intramuscular injection of different concentrations of rabies virus (8 × 10^4^ to 8 × 10^12^ copies) in to mice (10 mice per group) (A) is demonstrated. The melting curves and standard curves for rabies detection in the brain (B) (see method) was also demonstrated; Figure S2. The characteristics of wild-type (WT) and cGAS-/- mice injected with vehicle control or rabies virus at 7 days post-injection as indicated by fecal bacterial abundance in the class (A), order (B), family (top 15% highest abundance) (C), and genus (top 20% highest abundance) (D) are demonstrated (*n* = 3/group for WT ad cGAS-/- control, *n* = 6 for WT with rabies, and *n* = 7 for cGAS-/- with rabies).

## Abbreviations

The following abbreviations are used in this manuscript:

CNS	Central nervous system
TLR	Toll-like receptors
RIG-I	Retinoic acid-inducible gene I
cGAS	Cyclic GMP-AMP synthase
RNP	Ribonucleoprotein
mtDNA	Mitochondrial DNA
i.m.	Intramuscular
LPS	Lipopolysaccharide
BMMs	Bone-marrow-derived macrophages
dsRNA	Double-stranded RNA
