# Supplementary material for: Less Severe Inflammation in Cyclic GMP–AMP Synthase (cGAS)-Deficient Mice with Rabies, Impact of Mitochondrial Injury, and Gut–Brain Axis
Source: Biology (Basel). 2025 Nov 12;14(11):1583. doi: 10.3390/biology14111583 (PMC12650443; doi:10.3390/biology14111583)
Supplement: Supplementary file 1 [file biology-14-01583-s001.zip › Supplementary Material.pdf]

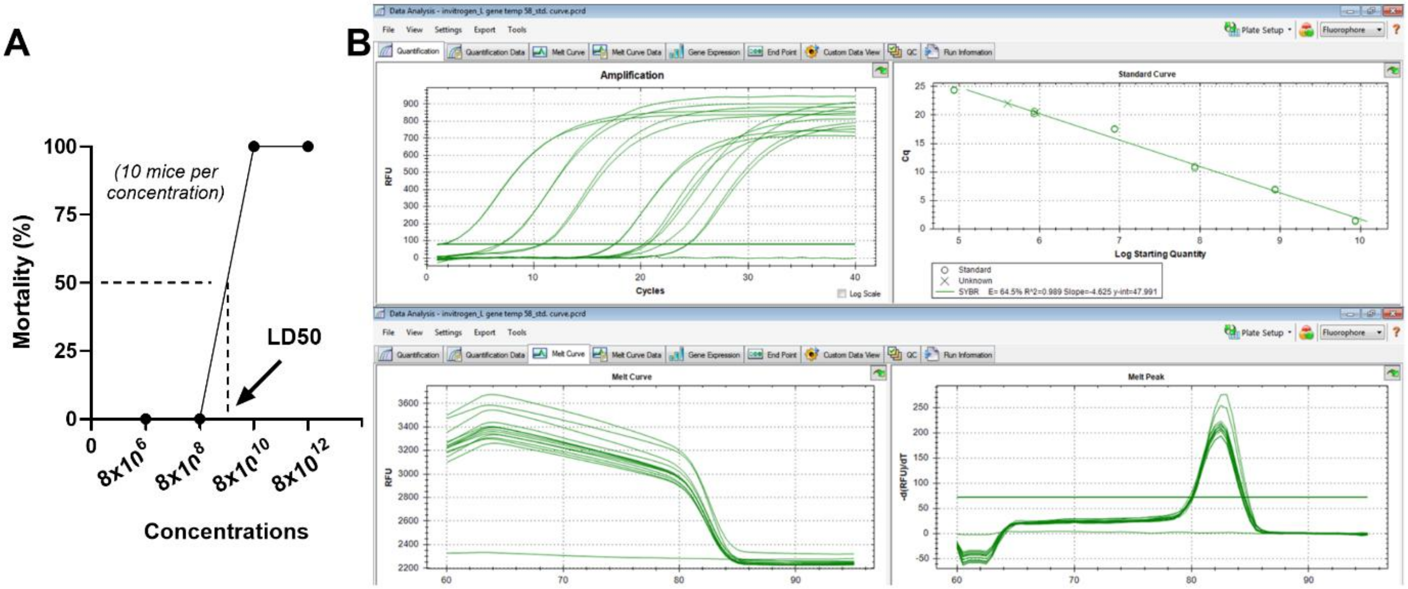

Supplement Figure S1. The determination of LD50 (Lethal Dose 50%) of CVS-11 rabies strain using intramuscular injection of different concentrations of rabies virus ($8x10^4$ to $8x\ 10^{12}$ copies) in to mice (10 mice per group) (A) is demonstrated. The melting curves and standard curves for rabies detection in the brain (B) (see method) was also demonstrated.

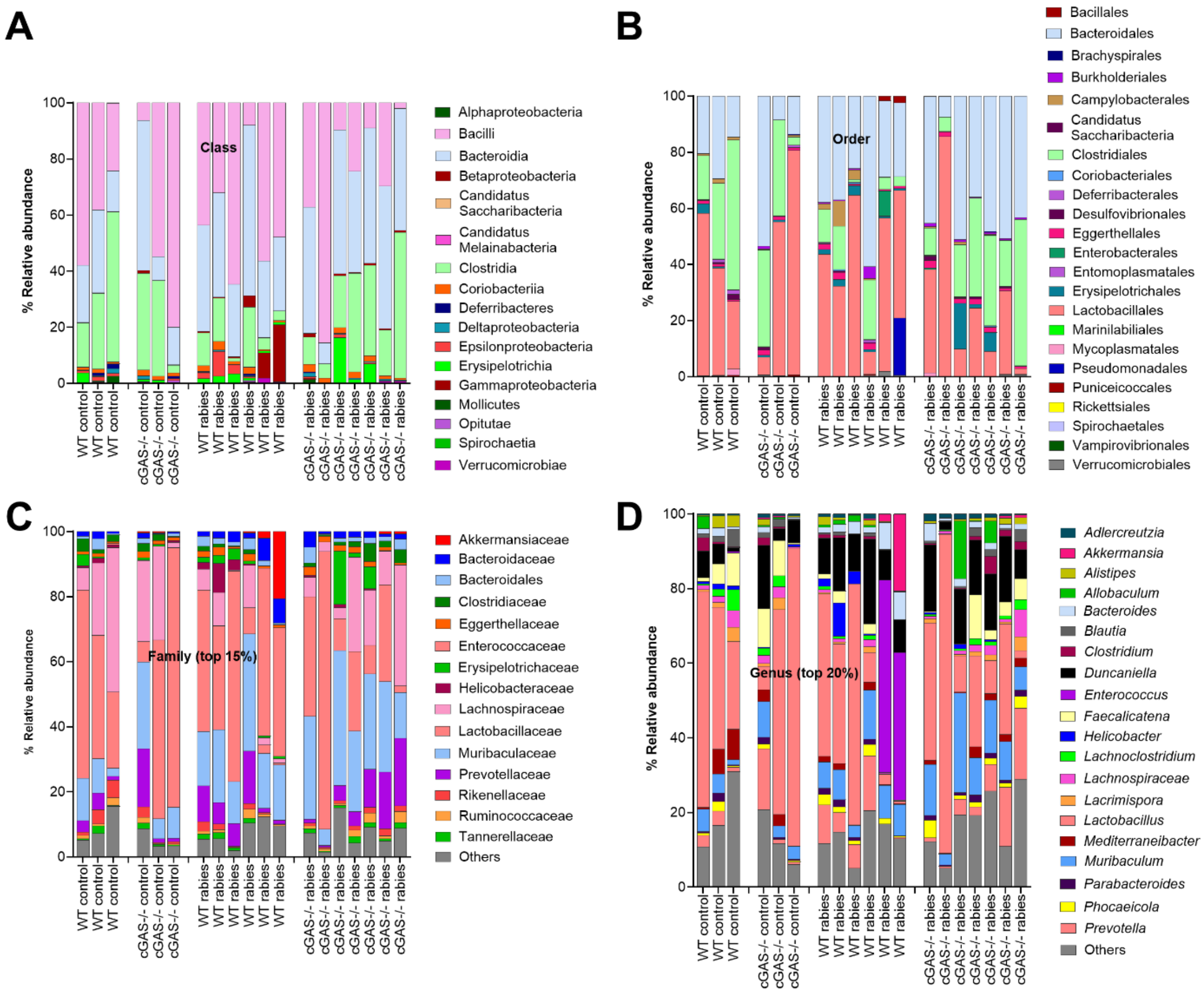

Supplement Figure S2. The characteristics of wild-type (WT) and cGAS-/- mice injected with vehicle control or rabies virus at 7 days post-injection as indicated by fecal bacterial abundance in the class (A), order (B), family (top 15% highest abundance) (C), and genus (top 20% highest abundance) (D) are demonstrated (n = 3/group for WT ad cGAS-/- control, n = 6 for WT with rabies, and n = 7 for cGAS-/- with rabies).
