# Supplementary figures and images for: Less Severe Inflammation in Cyclic GMP–AMP Synthase (cGAS)-Deficient Mice with Rabies, Impact of Mitochondrial Injury, and Gut–Brain Axis
Source: Biology (Basel). 2025 Nov 12;14(11):1583. doi: 10.3390/biology14111583 (PMC12650443; doi:10.3390/biology14111583)

The representative Western Blot analysis of Figure 6A

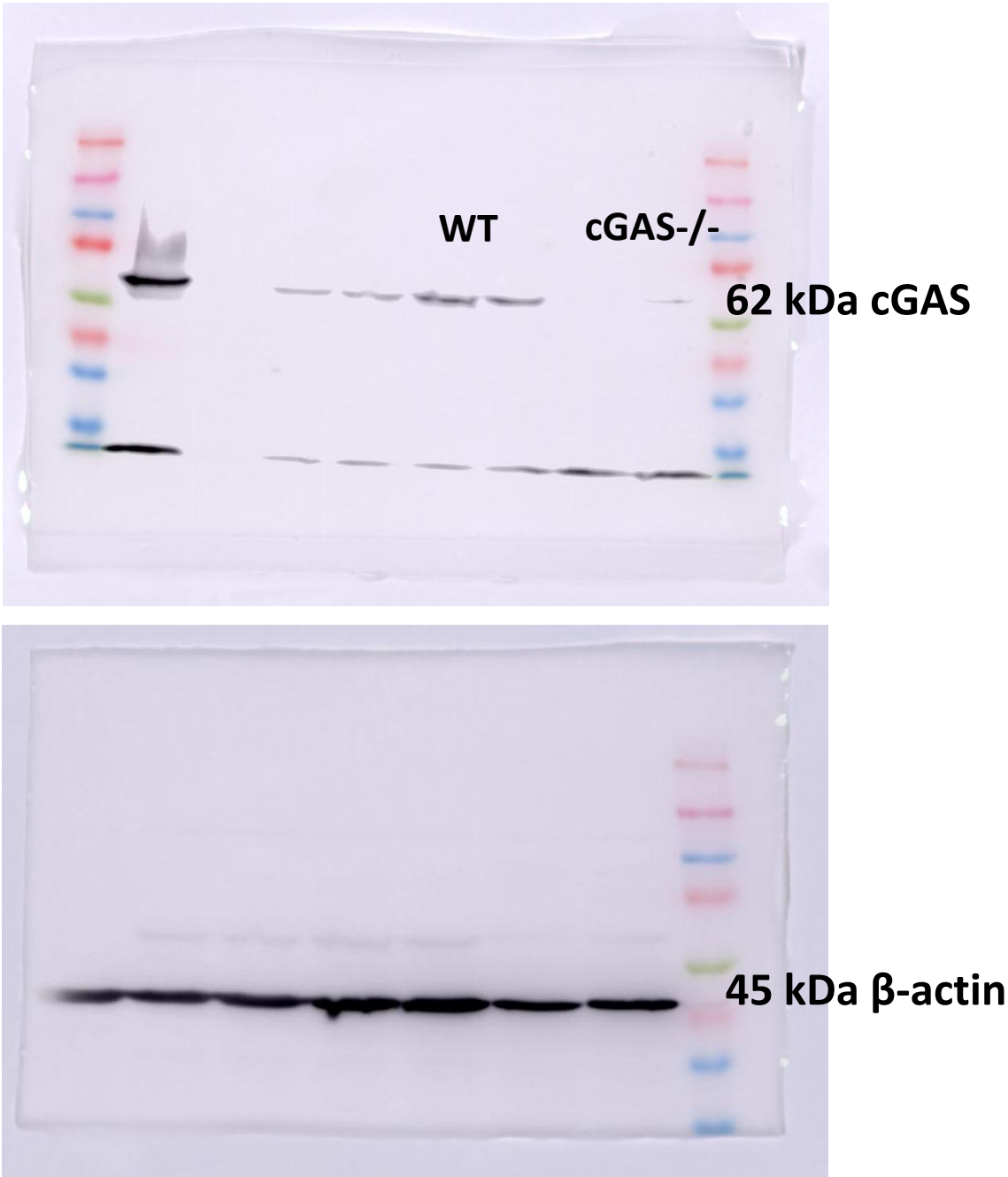

Supplement: Supplementary file 1 [file biology-14-01583-s001.zip › biology-3888385-uncropped Western Blot images.pdf]
